# Sequence of Eating at Japanese-Style Set Meals Improves Postprandial Glycemic Elevation in Healthy People

**DOI:** 10.3390/nu17040658

**Published:** 2025-02-12

**Authors:** Yuri Kurotobi, Hitoshi Kuwata, Mari Matsushiro, Yasuhiro Omori, Masahiro Imura, Susumu Nakatani, Miho Matsubara, Takuya Haraguchi, Shota Moyama, Yoshiyuki Hamamoto, Yuichiro Yamada, Yutaka Seino, Yuji Yamazaki

**Affiliations:** 1 Yutaka Seino Distinguished Center for Diabetes Research, Kansai Electric Power Medical Research Institute, Kyoto 553-0003, Japan; yuri.kurotobi@gmail.com (Y.K.); hitoshi.kuwa@gmail.com (H.K.); mr.matsushiro@gmail.com (M.M.); yasuhiro.omori.0303@gmail.com (Y.O.); imuramasahiro.kyoto@gmail.com (M.I.); susumu.nakatani@gmail.com (S.N.); m_jfr_0526@yahoo.co.jp (M.M.); t.haraguchi0819@gmail.com (T.H.); shota0531@gmail.com (S.M.); hamamoto.yoshiyuki@b4.kepco.co.jp (Y.H.); yamada.yuuichiro@a3.kepco.co.jp (Y.Y.); yutaka.seino.hp@gmail.com (Y.S.); 2 Center for Diabetes, Endocrinology and Metabolism, Kansai Electric Power Hospital, Osaka 553-0003, Japan

**Keywords:** meal sequence, incretin, diabetes, diet therapy, glucose monitoring

## Abstract

Background: The meal sequencing of macronutrients has been shown to ameliorate postprandial glucose excursion, but its effects in daily meals has not been investigated. We examined the impact on the glucose response to meal sequencing in healthy Japanese adults using continuous glucose monitoring (CGM) during a typical lunch meal. Methods: The test meal was a Japanese set meal or a beef and rice bowl, the contents of which were categorized as “rice” or “non-rice”. In the meal experiments, the subjects ingested the two categories of food in one of three orders: non-rice before rice, non-rice and rice together, and non-rice after rice. In the beef and rice bowl experiments, the subjects ingested either non-rice 15 min before rice or the two foods together. Results: The postprandial glucose level was measured over a 4 h period and the mean level of postprandial glucose was significantly lower than that when eating rice before non-rice or both together. Consuming non-rice before rice significantly reduced postprandial glycemic excursions in healthy adults in both experiments. Conclusions: Meal-sequencing by “eat carbs last” is a feasible dietary strategy for the better prevention and management of diabetes.

## 1. Introduction

Blood glucose levels are strictly regulated in the healthy individual under the sophisticated homeostatic system. The deterioration of the homeostasis such as the decrease in insulin secretory capacity and the increase in insulin resistance cause the impaired glucose tolerance manifesting as the persistent postprandial elevation of blood glucose begins in prediabetes. The postprandial glycemic spike in prediabetes, even before the sustained hyperglycemic state of diabetes, was already associated with an increased risk of cardiovascular disease and microvascular complications [1,2,3,4]. Early dietary intervention for prediabetes is advantageous for lowering the risk of diabetes and diabetic complications.

Postprandial glucose homeostasis is orchestrated by various factors, including nutrient quantity and composition, gastric emptying rate, the incretins glucagon-like peptide-1 (GLP-1) and glucose-dependent insulinotropic polypeptide (GIP), and insulin secretion [3,5,6]. Incretins are potent insulinotropic hormones secreted from the gut in response to nutrient loading: approximately two-thirds of the insulinotropic effect of incretins is elicited by GIP and one-third by GLP-1 [7]. GLP-1 and cholecystokinin, but not GIP, are reported to delay gastric emptying by regulating its rate according to the macronutrient content of food [8,9,10,11,12,13]. There are reports that the preloading of fat and protein enhances incretin secretion and delays gastric emptying [14,15,16,17] in advance of carbohydrate-induced glucose elevation. Meal sequencing thus presents an avenue to the mitigation of postprandial glycemic rises in healthy individuals as well as those with prediabetes for better health outcomes.

Meal sequencing for ameliorating the glycemic fluctuation has been reported for certain foods. The ingestion of whey protein or olive oil 30 min before mashed potato was reported to suppress glucose excursions [5,18]. In our earlier study, we demonstrated that ingesting meat or fish (protein and fat) 15 min before rice (carbohydrate) suppressed postprandial glucose elevation by promoting incretin secretion and reducing gastric motility [19]. However, neither the content nor the time intervals of the meals tested in previous studies could feasibly be directly applied to daily meals. There remains limited evidence to generalize the meal sequencing in an application of dietary therapy for healthy people or people with diabetes. To clarify the efficiency of meal sequencing for a daily meal, we investigated the effects of meal sequencing for a Japanese meal plate and for a rice bowl style, respectively, in mitigating postprandial glucose excursions by using continuous glucose monitoring (CGM).

## 2. Materials and Methods

### 2.1. Participants

Healthy volunteers between 20 and 50 years of age with a body mass index of 16–30 kg/m^2^ were enrolled. They do not have any regular illnesses and receive a company health checkup every year. Height, body weight, BMI, and blood samples at study entry were taken; blood glucose, glycated hemoglobin, total cholesterol, LDL cholesterol, HDL cholesterol, triglycerides, and standard indices of renal and hepatic function were determined. We excluded volunteers with allergic symptoms to the adhesives used in continuous glucose monitoring sensors, those with a history of gastrointestinal tract surgery, and those pregnant or potentially pregnant during the study period.

### 2.2. Ethics

The study was conducted at Kansai Electric Power Hospital, a single facility in Osaka, Japan, according to the principles of the Declaration of Helsinki. The protocol was approved by the Ethics Committee of Kansai Electric Power Hospital (UMIN registration number: UMIN000047131). Written informed consent was obtained from all participants.

### 2.3. Study Design

This study followed a randomized, crossover, non-blinded design. Participants wore continuous glucose monitoring devices (FreeStyle Libre, Abbott Laboratories, Chicago, IL, USA) for 14 days. A typical Japanese set meal (1913–2219 kJ, P 14.8–25.6 g; F 8.9–16.0 g; C 68.3–79.1 g) or a beef and rice bowl (2625 kJ, P 19.4 g; F 20.4 g; C 87.6 g) was employed as lunch. The meal contained 120 g steamed rice (796 kJ, P 3.0 g; F 0.4 g; C 44.5 g), a main dish, a side dish, soup, and dessert (fruit or gelatin), and was prepared in the cafeteria of Kansai Electric Power Hospital under the guidance of a dietitian. The participants were asked to gather at the cafeteria and eat there to check that the participants were following the correct order and timing for eating their meals, and that they had eaten everything. The food items included varied from day to day but were grouped as “rice” and “non-rice”. Non-rice included main dishes, side dishes, soup, and dessert. For the meal experiments, 5 meal sequences were as follows: (1) non-rice 15 min before rice (−15 dish), (2) non-rice 10 min before rice (−10 dish), (3) non-rice 5 min before rice (−5 dish), (4) whatever order participants preferred (together), and (5) non-rice 15 min after rice (+15 dish). There was no restriction on the order in which the non-rice elements were eaten. The beef and rice bowl contained 220 g steamed rice (1549 kJ, P 5.5 g; F 0.7 g; C 81.6 g) with simmered beef and onions on top (1076 kJ, P 13.9 g; F 19.7 g; C 6.0 g) and was grouped into “rice” and “non-rice”. The 2 meal sequences were as follows: (1) non-rice 15 min before rice (−15 beef) and (2) rice and non-rice consumed together (0 beef). The respective test meals were eaten at lunch on 7 separate days in random order after 4 h fast. The subjects’ CGM was scanned just before the test and the time to start the second course was set. The participants were monitored for compliance and maintained a normal level of physical activity for the duration of the study. The CGM-measured postprandial glucose concentrations over a 4 h period were collected; results are expressed as mean ± SD.

### 2.4. Calculation and Statistical Analyses

Incremental AUC of each measurement was calculated by subtracting the value of -the 15 min time point as a fasting glucose level before applying the trapezoidal rule.

Statistical analysis for the CGM traces was performed using Repeated Measures ANOVA. The analyses of Peak glucose and AUCs are indicated in the legends. *p*-value <0.05 was considered as statistically significant.

## 3. Results

Twenty-nine healthy volunteers were included in the study. The average age ± SD was 32.7 ± 6.6 years, BMI was 21.9 ± 2.8 kg/m^2^, and HbA1c was 5.2 ± 0.3%. The clinical characteristics are shown in Table 1. The daily activities and dietary habits of participants were asked to continue with their normal routine and fast for four hours before eating the test meals at lunch.

In the meal experiment, we primarily aimed to test whether eating rice last does efficiently suppress the postprandial glycemic rise regardless of the differences in menus. We also discussed how much time should be left between eating rice and non-rice food. The participants consumed a designated meal plate, the menus of which is changed daily, at lunch following the designated eating orders. The postprandial glycemic changes had monitored by CGM wore on at least two days before starting the experiment. Meal sequencing was examined by dividing the test meal into two parts, rice and non-rice elements, and whether the rice was eaten first, the non-rice elements first, and how long to wait before eating rice after starting to eat non-rice elements, as thoroughly written in the Method section. They followed their individual schedules for the meal sequencing, which means a randomized trial.

**Figure 1:**
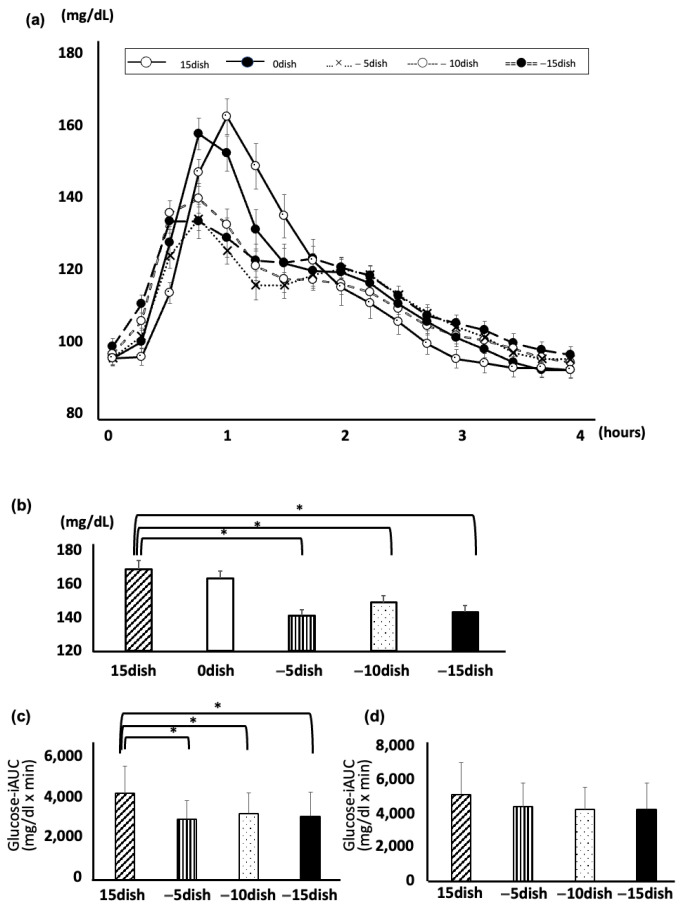
CGM measured postprandial glucose concentrations in the Japanese set meal. (**a**) Time course curves for glucose levels. Data are the mean ± standard error of the mean. Serving × time *p* < 0.05 by two-way repeated measures ANOVA. (**b**) Postprandial glucose peak. Two-way repeated measures ANOVA was used. Data are the mean ± standard error of the mean. * *p* < 0.05 vs. 15 dish. (**c**,**d**) The iAUC_0–120min_ (**c**) and the iAUC_0–240min_ (**d**) were indicated, respectively. The iAUCs were analyzed by one-way ANOVA with Bonferroni adjustment. * indicates *p* < 0.05 vs. 15 dish.

Twenty-six participants completed the protocol. The glucose levels of postprandial glucose over the four-hour period were significantly lower when non-rice was consumed before rice (−5 dish, −10 dish, and −15 dish) than when rice was consumed first (15 dish) or when rice and non-rice were consumed together (0 dish) (Figure 1a). The peak glucose levels were significantly lower when non-rice was consumed before rice (−5 dish, −10 dish, and −15 dish) than when rice was consumed first (15 dish): (−5 dish 141.3 ± 3.4 mg/dL; −10 dish 149.1 ± 3.8 mg/dL; −15 dish 143.3 ± 3.9 mg/dL; 15 dish 169.0 ± 5.1 mg/dL) (Figure 1b). There is a significant suppression of iAUCs_0–240min_ between “−10 dish” and “−15 dish” and “+15 dish”, while “−5 dish” did not reach statistical significance. On the other hand, the iAUCs_0–120min_ of all three groups (5 dish, −10 dish, and −15 dish) were significantly suppressed, compared with that of “+15 dish”, respectively. When comparing eating carbohydrates first or last in healthy subjects, the postprandial blood glucose rise was significantly higher when carbohydrates were eaten first. In addition, eating rice at 5, 10, or 15 min after starting to eat non-rice elements was shown to be superior to eating rice first in all cases, reducing the postprandial rise in blood glucose. This result suggested at least 5 min to wait for eating carbohydrates after start eating meals.

The beef and rice bowl experiment is designed to test the effectiveness of separating side dishes and rice as in a set meal rather than a rice bowl menu.

**Figure 2:**
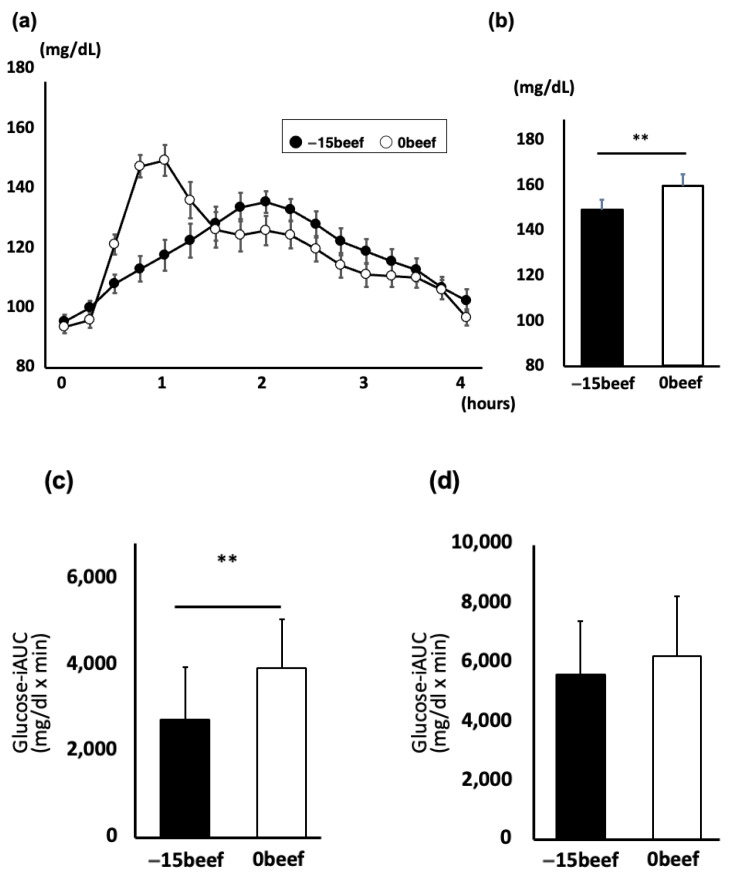
CGM-measured postprandial glucose concentrations in the beef bowl meal. (**a**) Time course curves for the glucose levels. Data are the mean ± standard error of the mean. Serving × time *p* < 0.05 by two-way repeated-measures ANOVA. (**b**) Postprandial glucose peaks in the beef and rice bowl experiment. Data are the mean ± standard error of the mean, which were analyzed by Paired *t* test. ** *p* < 0.01 vs. 0 beef. (**c**,**d**) The iAUC_0–120min_ (**c**) and the iAUC_0–240min_ (**d**) were indicated, respectively. The iAUCs were analyzed by paired *t* test vs. 0 beef. ** *p* < 0.01 vs. 0 beef.

The twenty-eight participants completed the protocol. The mean level of the postprandial glucose over four-hour period was significantly lower when non-rice was consumed before rice (−15 beef) than when rice and non-rice were consumed together (0 beef) (Figure 2a). The peak glucose level was significantly lower when the rice and beef plate was consumed separately (−15 beef) than when beef rice bowl (0 beef) was consumed (149.1 ± 4.5 mg/dL vs. 159.5 ± 5.1 mg/dL, *p* < 0.01, paired *t*-test), and the peak of postprandial glycemia was delayed in −15 beef compared to that in 0 beef (Figure 2b). The iAUC_0–120min_ of “−15 beef” was significantly suppressed, compared with that of “0 beef”, while the iAUCs_0–240min_ did not show a significant difference between the two. The results show that eating a set meal style, such as rice and side dishes, and eating rice last, rather than beef bowls as rice bowl menus, suppresses the post-meal rise in blood glucose. Interestingly, eating simmered beef before rice made the slope of glycemic rise milder than eating a beef rice bowl, which was not observed in the meal plate experiments. There is a distinction in the effects of meal sequencing within a menu or PFC content, which requires further study.

## 4. Discussion

In this study, we find that postprandial glucose excursion is suppressed when carbohydrate is consumed after protein and fat in a typical Japanese set meal in healthy adults, despite daily changes in the food content of the test meal. We also compared the ingestion of a beef and rice bowl when the beef was eaten before and with the rice and obtained results consistent with the meal experiment. These findings suggest that meal sequencing by consuming carbohydrates last in a daily meal can be beneficial for the management of blood glucose in a daily meal.

Interestingly, in a previous study of healthy adults, eating rice before meat (boiled chicken) and vegetables was found to elicit greater postprandial glucose excursions than when vegetables were included with the rice [20]. However, in that study, 180 g of leafy vegetable was used, which is an extraordinary condition, not a practical in the daily life. A previous study of prediabetics reported that eating chicken and vegetables for 10 min, followed by a 10 min rest interval and then eating bread (carbohydrates) elicited less post-meal glucose compared with the reverse order [21]. In our prior study, we reported similar findings using an interval between fish or meat and rice of 15 min [19]. Thus, a series of meal sequence experiments has consistently shown that eating non-carb elements first can suppress the elevation of postprandial blood glucose. However, these studies examined postprandial glucose when the interval between eating each category of food was 10–30 min [16,17,18,19,20,21,22,23,24], which is not appropriate for daily meals. We found similar improvement in this study using a more reasonable interval of 5 min between macronutrient ingestion to show that meal sequencing can be effective in daily life.

This study used continuous glucose monitoring to reveal the temporal dynamics of postprandial glucose fluctuations and provide compelling evidence of the benefit of meal sequencing in heathy individuals on a normal diet.

Even though the meal content varied in the test meal in the present study, consuming the non-rice category, a mixed protein and fat source, before the rice, a carbohydrate source, clearly mitigated postprandial glycemic elevations. Postprandial peak glycemia was similarly suppressed at −5 dish, −10 dish, and −15 dish in the test meal. With these findings, we can generalize the idea of meal sequencing on the postprandial glycemic elevation suggested by our previous data [19]. Interestingly, in the beef and rice bowl experiment, the postprandial increase in −15 beef was delayed compared to that of 0 beef, which was not the case in the meal experiment. This may be because the beef and rice bowl contained more fat than the meal, and fat delays gastric emptying. On the other hand, the iAUCs_0–240min_ did not show a significant change, suggesting that the fall in blood glucose might be prolonged. Further study is required to confirm this. The strength of this study is that we used typical dietary content and more modest intervals that better simulate ordinary eating behavior to show that meal sequencing is both beneficial and feasible in daily life. A limitation of this study is that blood glucose was only determined by CGMS. We focused on a 4 h postprandial period, but there is a possibility that the extended glucose fluctuations beyond this time frame were missed. In addition, a test meal prepared in a hospital setting was different day to day, although the total calorie and PFC balance were matched. To evaluate whether the effect of meal sequencing is effective in other cuisines, further investigation is required. In conclusion, meal-sequencing by “eat carbs last” in a typical Japanese meal plate improves postprandial glycemic excursions in healthy adults.

## Figures and Tables

**Table 1:** Characteristics of participants. Data are shown as mean ± standard deviation. HbA1c, hemoglobin A1c.

Characteristics of Participants	
n (male/female)	29 (21/8)
Age (years)	32.7 ± 6.6
BMI (kg/m^2^)	21.9 ± 2.8
Fasting plasma glucose (mg/dL)	92.8 ± 9.1
HbA1c (%)	5.2 ± 0.3

Data are shown as mean ± standard deviation. HbA1c, hemoglobin A1c.

## Data Availability

The data presented in this study are available on request from the corresponding author on reasonable request.
